# Olanzapine-induced hepatopathy in albino rats: A newer model for screening putative hepatoprotective agents, namely silymarin

**DOI:** 10.4103/0253-7613.71924

**Published:** 2010-12

**Authors:** Parama Sengupta, Chiranjib Bagchi, Abhishek Sharma, G. Majumdar, C. Dutta, Santanu Tripathi

**Affiliations:** Department of Pharmacology, Burdwan Medical College & Hospital, Burdwan, West Bengal, India

**Keywords:** Olanzapine, hepatopathy, silymarin

## Abstract

**Backgrounds:**

This study was conducted to establish olanzapine-induced hepatopathy in Wistar albino rats as a newer model to screen putative hepatoprotective agents namely silymarin.

**Materials and Methods:**

Albino rats were divided into three groups, namely vehicle control group (CG), olanzapine-treated group (OZ), and olanzapine plus silymarin (OZS) treated groups. Both the OZ and OZS groups were treated with the same dose of intraperitoneal olanzapine for 6 weeks and group OZS additionally received oral silymarin. Baseline and terminal hepatic enzymes (SGOT, SGPT, and ALP) were measured in all three groups.

**Results:**

Histopathological examination of livers of both OZ and OZS groups showed degenerative changes, whereas those of control group showed normal architecture. Liver enzyme levels showed statistically significant rise in comparison to the control group as well as the respective base line values in both the test groups, but the differences in the rise of liver enzymes between the two test groups were not statistically significant.

**Conclusion:**

Olanzapine-induced hepatopathy in rats can be used as a model for screening putative hepatoprotective agents and in our setting silymarin has failed to provide any hepatoprotection.

## Introduction

Olanzapine is a widely used atypical antipsychotic agent, approved by the U.S. Food and Drug Administration for bipolar disorder and schizophrenia.[1] Although the general consensus is that hepatic side effects are not so common with the use of antipsychotic agents, reports of olanzapine-induced hepatic damage have been published both from animal experiments as well as clinical studies.[2–5] Various chemicals (e.g., alcohol, CCl_4_, alcohol–CCl_4_, etc.) and drugs (paracetamol, nimesulide, etc.) are used for screening of hepatoprotective agents. Silymarin is a polyherbal preparation obtained from “milk thistle” (*Silybum marianum*) and is mostly used for hepatoprotection.[6] In this study, we aim to establish olanzapine-induced hepatopathy in Wistar albino rats as a newer model to screen putative hepatoprotective agents namely silymarin.

## Materials and Methods

The study was conducted after obtaining permission from the Institutional Animal Ethics Committee. Nine Wistar albino rats (200–210 g) were procured from the institutional animal house. The rats were housed at the institutional animal house in standard plastic cages in an air-conditioned room at 22 ± 1°C under controlled lighting (14 h light/10 h dark cycle). Standard rat food and tap water *ad libitum* were given. All the procedures were performed in accordance with the CPCSEA (Committee for the Purpose of Control and Supervision on Experiments on Animals) guidelines.

The rats were divided into three groups olanzapine-treated group (OZ) (three rats), olanzapine plus silymarin-treated groups (OZS) (three rats), and vehicle-treated control group (CG) (three rats). Rats of OZ group received olanzapine (Tablet OLEXA, CIPLA) i.p. at a rate of 4 mg/kg dissolved in 0.9% saline daily for a period of 6 weeks. OZS group received the same amount of olanzapine i.p. for 6 weeks along with silymarin at a dose of 200 mg/kg orally daily for the same duration and the rats of the control group received 0.9% saline i.p. as the OZ and OZS groups. For this study, olanzapine dosage was chosen as equivalent to the highest dosages used in humans (0.5 mg/kg/day).[5] Silymarin (SYRIAL Syrup, OLCARE LAB, 35 mg/5 mL) was given at a dose of 200 mg/kg.[7] Liver enzymes SGPT, SGOT, and ALP levels were measured prior to any drug therapy, i.e., at baseline as well as at the end of the study, i.e., after 6 weeks. At the end of the study, all the rats were killed humanely, livers were dissected, and transferred in 10% formal saline solution for histopathological examination. The sections of hepatic tissue were stained with both hematoxyline–eosin (H and E) stain as well as reticulin stain (Gordon and Sweet’s method).

Within groups, comparisons were done using the paired *t*-test, and between groups comparison were done using unpaired *t*-test.

## Results

The hepatic enzyme (SGOT, SGPT, and ALP) levels showed no baseline variability among the three groups. All the hepatic enzyme levels of group CG, OZ, and OZS are depicted in Tables 1–3.

**Table 1:** Hepatic enzyme levels in control group (CG)

*Statistical parameters*	*CG*					
	*SGPT*		*SGOT*		*ALP*	
	*BL*	*6 weeks*	*BL*	*6 weeks*	*BL*	*6 weeks*
Mean ± SD	39.5 ± 2	40.16 ± 1.75	28 ± 2	29 ± 2.64	143.5 ± 3.05	144 ± 2.08
Median	28	40	28	28	145	145
Maximum	36	38.5	30	32	145.5	146
Minimum	30	42	26	27	140	141
Interquartile range (Q3 – Q1)	29 – 27 = 2	41 – 39.25 = 1.75	29 – 27 = 2	30 – 27.5 = 2.5	145.25 – 142.5 = 2.75	145.5 – 143 = 2.5

**Table 2:** Hepatic enzyme levels in olanzapine-treated group (OZ)

*Statistical parameters*	*OZ*					
	*SGPT*		*SGOT*		*ALP*	
	*BL*	*6 weeks*	*BL*	*6 weeks*	*BL*	*6 weeks*
Mean ± SD	40.33 ± 1.52	165.33 ± 3.39	28 ± 2.64	52.667 ± 4.17	145.33 ± 3.05	178 ± 1
Median	40	164	27	48	146	177
Maximum	42	170	31	56	148	179
Minimum	39	162	26	48	142	177
Interquartile range (Q3 – Q1)	41 – 39.5 = 1.5	167 – 163 = 4	29 – 26.5 = 2.5	55 – 51 = 4	148 – 142 = 6	179 – 177 = 2

**Table 3:** Hepatic enzyme levels in olanzapine plus silymarin-treated group (OZS)

*Statistical parameters*	*OZS*					
	*SGPT*		*SGOT*		*ALP*	
	*BL*	*6 weeks*	*BL*	*6 weeks*	*BL*	*6 weeks*
Mean ± SD	41 ± 2.64	155.33 ± 2.645	27.33 ± 2.08	55.66 ± 3.78	145.33 ± 3.05	174.33 ± 2.08
Median	40	157	28	54	146	175
Maximum	44	158	29	60	148	176
Minimum	39	153	25	53	142	172
Interquartile range (Q3 – Q1)	42 – 39.5 = 2.5	157.5 – 155 = 2.5	28.5 – 26.5 = 2	57 – 53.5 = 3.5	147 – 144 = 3	175.5 – 173.5 = 2

**Chart 1:**
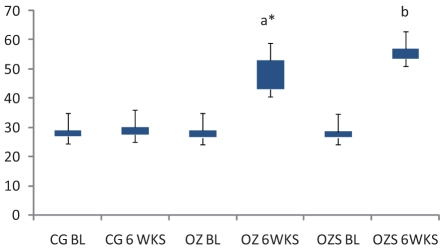
SGOT level in all three groups. Legend: CG, control group; OZ, olanzapine-treated group; OZS, olanzapine plus silymarin-treated group; BL, base line; 6WKS, 6 weeks; a and b—*P* values when compared with corresponding base line values, a—*P* = 0.045, b—*P* = 0.091.

**Chart 2:**
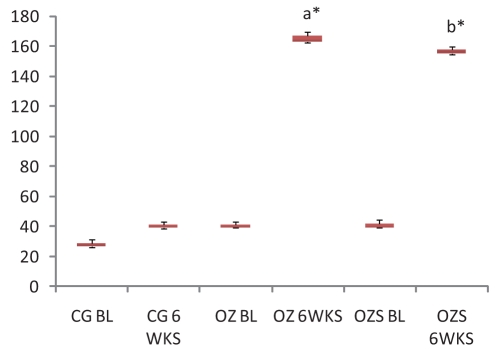
SGPT level in all three groups. Legend: CG, Control group; OZ, olanzapine-treated group; OZS, olanzapine plus silymarin-treated group; BL, base line; 6WKS, 6 weeks; a and b—*P* values when compared with corresponding base line values, a—*P* = 0.045, b—*P* = 0.001.

**Chart 3:**
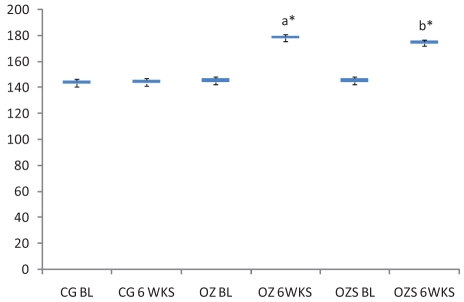
ALP level in all three groups. Legend: CG, control group; OZ, olanzapine-treated group; OZS, olanzapine plus silymarin-treated group; BL, base line; 6WKS, 6 weeks, a and b—*P* values when compared with corresponding base line values, a—*P* = 0.004, b—*P* = 0.007.

**Figure 1:**
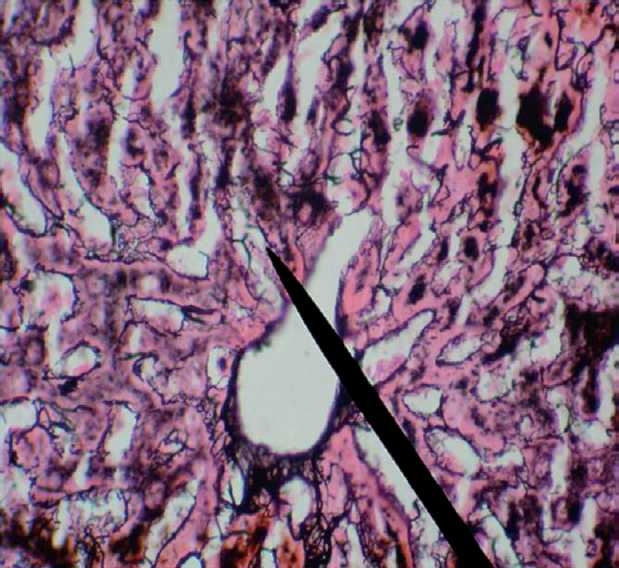
Reticulin stained hepatic section of rat of OZ group shows degenerative changes as well as vascular congestion, 400X.

**Figure 2:**
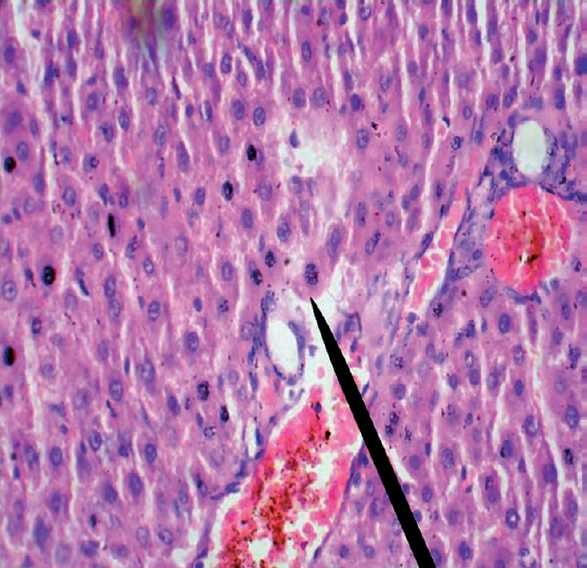
H & E stained hepatic section of rat of OZS group shows degenerative changes along with karyorrhexis, 400X.

**Figure 3:**
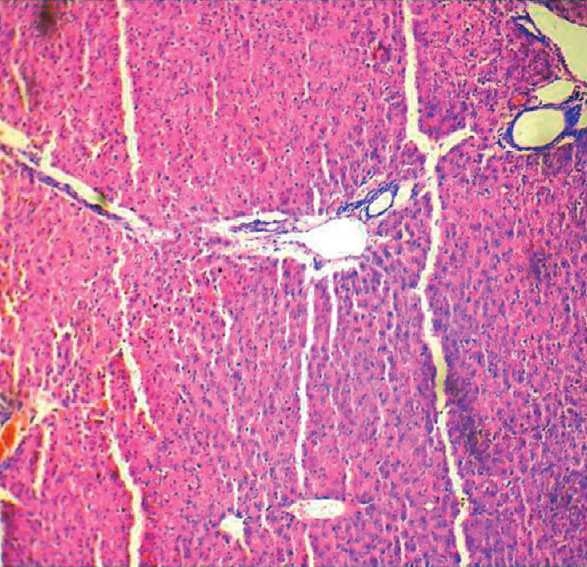
H & E stained hepatic section of rat of CG group show normal architecture, 100X.

On comparison with the baseline values, at 6 weeks, all the three hepatic enzymes (SGOT, SGPT, and ALP) were raised significantly in the OZ group (SGOT, *P* = 0.045; SGPT, *P* = 0.000, and ALP, *P* = 0.004). In OZS group, when hepatic enzyme levels were compared with the corresponding baseline values, the rise in SGPT (*P* = 0.001) and ALP (*P* = 0.007) levels were significant, but the rise in SGOT (*P* = 0.091) level was not significant (Charts 1–3). When compared individually with the CG group, a statistically significant rise in the hepatic enzymes levels both in the OZ group (SGOT, *P* = 0.028; SGPT and ALP, *P* = 0.000) and OZS group (SGOT, *P* = 0.046, SGPT, and ALP, *P* = 0.000) were seen. However, on comparison between OZ and OZS groups, the rise was not statistically significant (SGOT, *P* = 0.0779; SGPT, *P* = 1.01; and ALP, *P* = 0.051), thereby showing the failure of silymarin to provide any protection against olanzapine-induced liver damage. The hepatic sections of rats of both the OZ and OZS groups showed ballooning degeneration as well as vascular congestion [Figures 1 and 2, respectively] in comparison to normal hepatic architecture in control group [Figure 3]. In some sections, nuclear fragmentations, i.e., features of karyorrhexis were also evident [Figure 2].

## Discussion

Although there are various screening models which are employed to assess the efficacy of hepatoprotective agents, olanzapine-induced hepatopathy in albino rats can be used as a model to screen putative hepatoprotective agents. Silymarin, a polyphenolic flavonoid derived from milk thistle, acts by inhibiting lipid peroxidation. It helps to reduce or prevent liver damage caused by alcohol, poisonous mushrooms, drugs, and other toxins.[6] However, in this study it has failed to offer any protection against olanzapine-induced liver damage both biochemically as well as histopathologically.

A small sample size and unexplored role of other hepatoprotective agents on this model remain the limitations of this study. Therefore, further experimentations with other hepatoprotective agents including silymarin using more number of animals are required to establish the validity of olanzapine-induced hepatopathy model in Wistar albino rats for screening putative hepatoprotective agents.
